# Long-Segment Right Colon Volvulus in a Young Adult With Severe Developmental Disability: An Unusual Presentation and a Diagnostic Challenge

**DOI:** 10.7759/cureus.102936

**Published:** 2026-02-03

**Authors:** Luu N Pham, Jericho Ghanem, Troy Kerner

**Affiliations:** 1 Surgery, University of New England College of Osteopathic Medicine, Portland, USA; 2 Surgery, Lower Bucks Hospital, Bristol, USA

**Keywords:** bird’s beak sign, cecal volvulus, chronic constipation, colonic malfixation, contrast enema, developmental disability, pseudo-obstruction, right colon volvulus, toxic megacolon mimic, transverse colon

## Abstract

A 22-year-old woman with severe developmental disability, percutaneous endoscopic gastrostomy dependence, and chronic constipation presented with progressive abdominal distension and obstipation. Initial abdominal radiography demonstrated diffuse gaseous distension without a clear transition point, and she was discharged after symptomatic management. She returned within 24 hours with worsening distension, tachycardia, leukocytosis, and an elevated lactate level. Computed tomography of the abdomen and pelvis demonstrated marked dilation of the right colon with a maximal diameter of approximately 13 cm, along with pronounced cephalad displacement and organ shift, raising concern for toxic megacolon or acute colonic pseudo-obstruction. A water-soluble contrast enema demonstrated abrupt tapering at the distal transverse colon with a classic bird’s beak configuration, consistent with mechanical torsion. Urgent exploratory laparotomy confirmed a long-segment right-colon volvulus involving the cecum, ascending colon, and proximal transverse colon with obstruction at the mid-transverse colon. The patient underwent decompression and right hemicolectomy with primary ileocolic anastomosis and recovered without complication.

## Introduction

Colonic volvulus refers to the torsion of a segment of the colon around its mesentery, resulting in luminal obstruction and potential vascular compromise [1]. Although colonic redundancy may predispose to volvulus, acquired factors such as chronic constipation and colonic dysmotility also contribute to it [1,2]. Sigmoid volvulus accounts for most cases and predominantly affects older adults, whereas cecal volvulus is less common and more frequently occurs in younger patients in the United States [3]. Extension of a cecal or right colon volvulus into the transverse colon is exceedingly rare and can pose diagnostic challenges when imaging findings overlap with toxic megacolon or acute colonic pseudo-obstruction [4]. Colonic perforation represents the most serious complication and can lead to peritonitis, sepsis, and death, necessitating prompt surgical intervention when ischemia or gangrene is suspected [1,3,5]. Because volvulus may involve different segments of the gastrointestinal tract, extensive bowel resection may be required with potentially life-altering consequences [6,7]. We report an unusual long-segment right colon volvulus causing mechanical obstruction at the mid-transverse colon in a medically complex young adult with marked cephalad displacement of the dilated colon.

## Case presentation

A 22-year-old woman with a complex neurodevelopmental history, including agenesis of the corpus callosum, neuromuscular scoliosis, and severe intellectual disability, presented with progressive abdominal distension and obstipation. She was nonverbal, wheelchair-bound, and fully dependent for activities of daily living. She received all nutrition via a percutaneous endoscopic gastrostomy (PEG) tube with feeds administered three times daily and had chronic constipation managed intermittently with enemas. Caregivers noted that her stools, typically loose, had recently become more formed. Over approximately 48 hours, she developed worsening abdominal distension and cessation of stool output. At home, PEG decompression yielded bilious output without symptomatic relief, and an enema produced only a small bowel movement, prompting emergency department evaluation.

**Figure 1:**
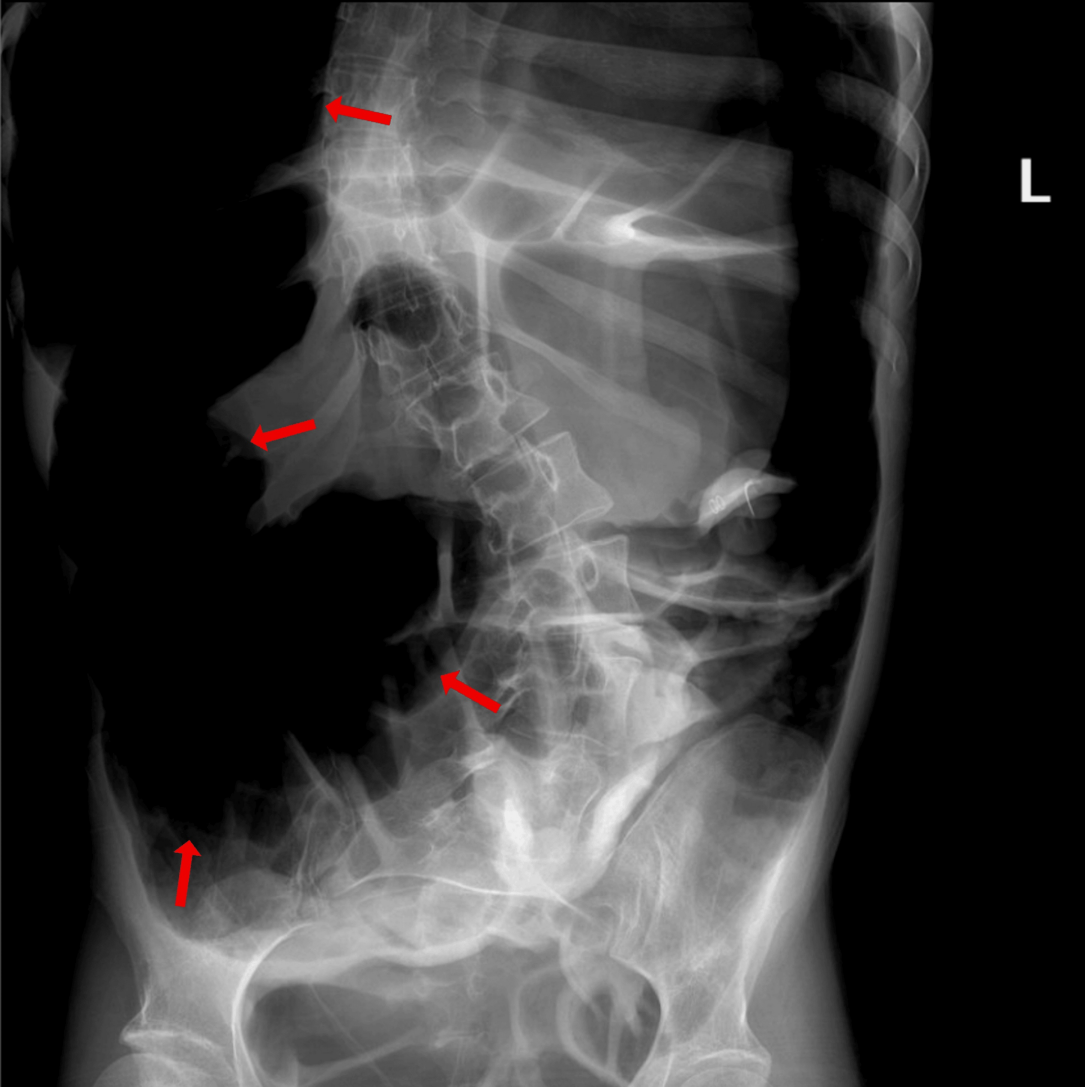
Initial abdominal X-ray demonstrating diffuse gaseous bowel distention with possible megacolon Supine anteroposterior abdominal radiograph from the initial emergency department visit shows widespread gas throughout the small and large bowel (arrows). The colon reaches a maximal diameter of approximately 8 cm, raising concern for ileus or megacolon. No fecal loading or free intraperitoneal air is seen.

At her initial visit, she was hemodynamically stable and laboratory studies were unremarkable. Abdominal radiography demonstrated diffuse gaseous distension of the small and large bowel with a maximal colonic diameter of approximately 8 cm, without fecal loading or free intraperitoneal air (Figure 1).

These findings were interpreted as ileus or early megacolon, and she was discharged with close monitoring instructions.

**Figure 2:**
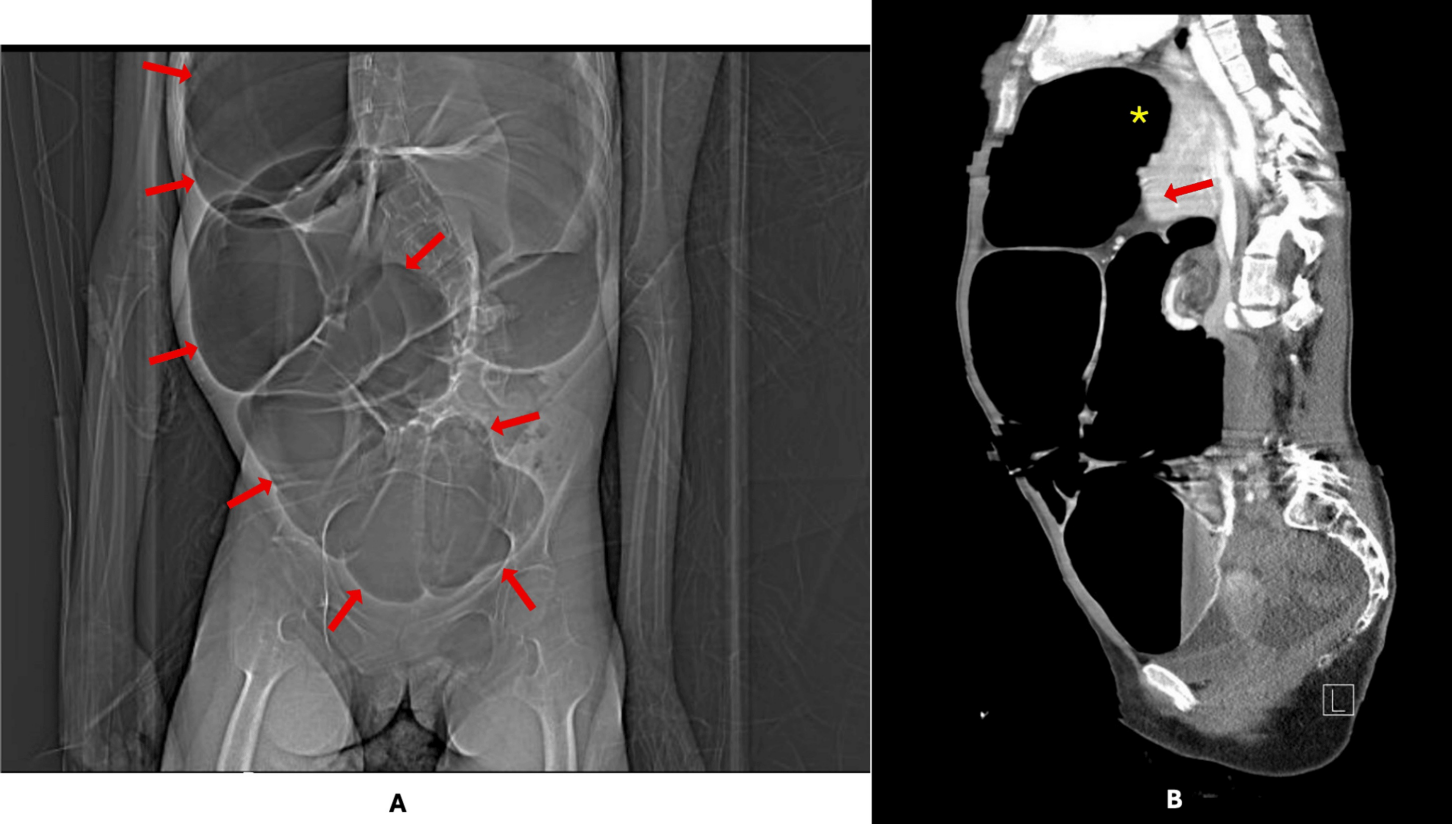
Computed tomography of the abdomen and pelvis with intravenous contrast on hospital day zero (A) Coronal image demonstrating marked dilation of the cecum and ascending colon with gaseous distension (arrows) occupying much of the abdominal cavity, with a maximal diameter of approximately 13 cm. (B) Sagittal image showing extreme cephalad displacement of the dilated right colon into the right hemithorax (asterisk), elevation of the right hemidiaphragm, and medial displacement of the liver (arrow).

Within 24 hours of discharge, she re-presented with worsening abdominal distension and persistent hiccups. She remained afebrile but was tachycardic, with a markedly distended yet soft abdomen and exaggerated bowel sounds. PEG feeds were stopped and the tube was placed to gravity drainage. Repeat laboratory evaluation demonstrated worsening leukocytosis (white blood cell count 9.8 to 12.7 ×10³/µL; reference interval 4.5-11.0 ×10³/µL), rising blood urea nitrogen (17 to 26 mg/dL; reference interval 7-20 mg/dL), and increasing lactate (1.2 to 2.5 mmol/L; reference interval 0.5-2.2 mmol/L). These changes were observed across serial measurements, from the initial and repeat evaluations over approximately 38 hours. Computed tomography of the abdomen and pelvis with intravenous contrast demonstrated marked dilation of the right colon (cecum and ascending colon) occupying much of the abdominal cavity, with a maximal diameter of approximately 13 cm (Figure 2A).

Additional findings included pronounced cephalad displacement, elevation of the right hemidiaphragm, medial displacement of the liver, and free pelvic fluid (Figure 2B). The initial radiologic impression raised concern for possible toxic megacolon, and a contrast enema was recommended for further characterization.

**Figure 3:**
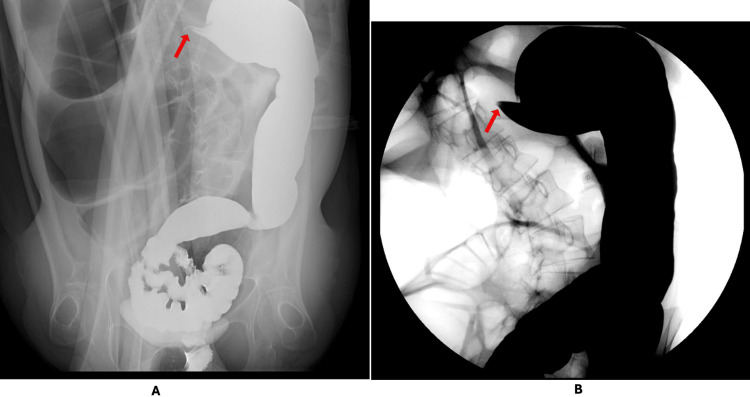
Water-soluble contrast enema on hospital day one (A) Abdominal radiograph showing contrast opacification of the colon with abrupt narrowing at the distal transverse colon (arrow). (B) Fluoroscopic image demonstrating an abrupt bird’s beak taper at the distal transverse colon (arrow), consistent with mechanical torsion.

A bedside attempt at rectal decompression using a 22-French red-rubber catheter produced no air or stool. A water-soluble contrast (Gastrografin, Schering AG, Berlin, Germany) enema demonstrated brisk passage of contrast from the rectum through the descending colon, followed by abrupt tapering and termination at the distal transverse colon with a classic bird’s beak appearance, consistent with a mechanical point of torsion (Figure 3).

At the initial presentation, the diffuse colonic distension without a clear transition point supported consideration of ileus or early megacolon. On re-presentation with worsening distension and tachycardia, acute colonic pseudo-obstruction, and toxic megacolon were considered; however, the absence of diarrhea or systemic toxicity made toxic megacolon less likely. The contrast enema findings confirmed a mechanical obstruction, establishing the diagnosis of a long-segment right colon volvulus involving the cecum, ascending colon, and proximal transverse colon.

Given the severe right-sided colonic distension, the discrete mechanical transition point on contrast enema, and concern for impending perforation in the setting of rising leukocytosis and lactate, urgent exploratory laparotomy was recommended. Written consent was obtained from the patient’s legal guardian. Under general anesthesia, a midline laparotomy revealed a massively distended colon immediately beneath the peritoneum. Exploration demonstrated marked dilation of the cecum, ascending colon, and proximal transverse colon with a long-segment right-colon volvulus causing torsion and mechanical obstruction at the mid-transverse colon. The right-colon mesentery was thin and highly mobile with minimal retroperitoneal fixation, consistent with malfixation. No perforation was present, and the remaining bowel appeared viable.

A decompressive colotomy was performed to facilitate exposure and was subsequently oversewn. A right hemicolectomy was then performed with resection from the terminal ileum through the proximal transverse colon, followed by a side-to-side stapled ileocolic anastomosis with reinforcement sutures and closure of mesenteric defects. Estimated blood loss was less than 50 mL. Postoperatively, the patient was managed with intravenous fluids, PEG-to-gravity drainage, ceftriaxone and metronidazole, acid suppression, multimodal analgesia, and close electrolyte monitoring.

By postoperative day one, she was clinically stable with improving leukocytosis and correction of electrolyte abnormalities. A transient postoperative ileus resolved by postoperative day two, allowing resumption of PEG feeds without recurrence of symptoms. Surgical pathology was consistent with right colon volvulus and correlated with operative findings, without evidence of ischemia or malignancy. She was discharged on postoperative day seven at her baseline functional status with normal bowel function and planned outpatient follow-up.

## Discussion

This case highlights several clinically important features of right colon volvulus in a medically complex young adult, including an uncommon long-segment volvulus extending into the proximal transverse colon with mechanical obstruction at the mid-transverse colon, imaging findings that mimicked toxic megacolon and contributed to diagnostic uncertainty, and the decisive role of water-soluble contrast enema when computed tomography findings and clinical context were discordant.

A right colon volvulus occurs when a mobile right colon twists around its mesentery (axial cecal volvulus) or folds anterosuperiorly (cecal bascule), most often due to congenital non-fixation or malrotation [1-3]. Acquired factors such as chronic constipation, intestinal dysmotility, distal obstruction, and prior abdominal procedures may precipitate torsion and contribute to more complex surgical presentations in anatomically predisposed patients [2,3,6]. In this case, chronic constipation, prolonged immobility, and intraoperative evidence of minimal retroperitoneal fixation with a thin, highly mobile mesentery likely contributed to progressive colonic distension and vulnerability to torsion [6].

Diagnosis of right colon volvulus can be challenging. Plain abdominal radiography may show diffuse gaseous distension without a clear transition point, leading to misdiagnosis as acute colonic pseudo-obstruction or toxic megacolon [8]. Computed tomography is valuable for defining the distribution of colonic dilation and associated organ displacement, but findings may overlap with nonmechanical causes of megacolon. In this case, marked right-sided dilation with pronounced cephalad displacement raised initial concern for toxic megacolon despite the absence of colitis, systemic toxicity, or diarrhea. Given the discordance between the CT impression and the clinical context, along with worsening abdominal distension and objective deterioration across serial measurements (lactate 1.2 to 2.5 mmol/L; leukocytosis 9.8 to 12.7 ×10³/µL), a water-soluble contrast enema was pursued to evaluate for mechanical obstruction and localize a transition point. The resulting bird’s beak configuration at the distal transverse colon confirmed mechanical torsion and expedited definitive operative management [1,2].

Management of right colon volvulus differs from sigmoid volvulus. Nonoperative decompression, which can be effective for sigmoid volvulus, is less successful for cecal or right colon volvulus and may increase the risk of perforation in a markedly distended bowel [2]. Definitive surgical management, most commonly right hemicolectomy, is recommended to prevent recurrence and catastrophic complications when volvulus is suspected or confirmed [1-3]. Early operative intervention in this patient allowed definitive treatment prior to ischemia or perforation, enabling primary anastomosis and an uncomplicated recovery.

The patient’s neuromuscular scoliosis is also relevant to the atypical imaging appearance. While the intraoperative finding of a thin, highly mobile right colon mesentery with minimal retroperitoneal fixation is most consistent with congenital malfixation rather than a direct consequence of scoliosis, severe spinal deformity may indirectly amplify risk by altering intra-abdominal geometry, limiting mobility, and worsening chronic constipation and progressive distension [9]. In this context, scoliosis may have contributed to the exaggerated organ displacement and extreme cephalad positioning of the dilated colon (Figure 2), producing an appearance on chest radiography that was initially interpreted as right lung collapse. Severe abdominal distension can elevate the hemidiaphragm and compress the lung base, thereby mimicking primary thoracic pathology. Correlation with abdominal imaging is essential to avoid misinterpretation and unnecessary pulmonary evaluation [10,11].

## Conclusions

In chronically constipated, immobile, medically complex patients, right colon volvulus should remain a diagnostic consideration, even in younger adults and when initial radiographs are nonspecific. This case highlights an uncommon long-segment right colon volvulus with an atypical transition point at the distal to mid-transverse colon, demonstrating that right-sided volvulus may deviate from classic anatomic expectations and present with misleading imaging findings. Marked right-sided colonic dilation with pronounced cephalad displacement and organ shift can mimic toxic megacolon or acute colonic pseudo-obstruction, particularly when a discrete transition point is not evident early, and the clinical picture is discordant with colitis. In patients with severe neuromuscular scoliosis, altered intra-abdominal geometry and chronic immobility may further accentuate organ displacement and distension, increasing diagnostic difficulty. In this setting, a water-soluble contrast enema is a practical, rapid adjunct to clarify the etiology, localize a mechanical obstruction, and confirm torsion, thereby expediting definitive management. Prompt operative intervention is essential to prevent ischemia and perforation. When bowel viability is preserved, definitive resection with primary anastomosis can provide durable treatment with a low risk of recurrence.
